# Facile and Low-Cost SPE Modification Towards Ultra-Sensitive Organophosphorus and Carbamate Pesticide Detection in Olive Oil

**DOI:** 10.3390/molecules25214988

**Published:** 2020-10-28

**Authors:** Dionysios Soulis, Marianna Trigazi, George Tsekenis, Chrysoula Chandrinou, Apostolos Klinakis, Ioanna Zergioti

**Affiliations:** 1 Biomedical Research Foundation of the Academy of Athens, Soranou Ephessiou 4, 11527 Athens, Greece; dsoulis1@gmail.com (D.S.); mtrigazi@gmail.com (M.T.); gtsekenis@bioacademy.gr (G.T.); aklinakis@bioacademy.gr (A.K.); 2 School of Mathematical and Physical Sciences, Physics Department, National Technical University of Athens, Heroon Polytehneiou 9, 15780 Athens, Greece; cchandr@mail.ntua.gr

**Keywords:** pesticide, carbamate, carbofuran, organophosphorus, chlorpyrifos, carbon black, acetylcholinesterase, LIFT, electrochemical biosensor, olive oil

## Abstract

Despite the fact that a considerable amount of effort has been invested in the development of biosensors for the detection of pesticides, there is still a lack of a simple and low-cost platform that can reliably and sensitively detect their presence in real samples. Herein, an enzyme-based biosensor for the determination of both carbamate and organophosphorus pesticides is presented that is based on acetylcholinesterase (AChE) immobilized on commercially available screen-printed carbon electrodes (SPEs) modified with carbon black (CB), as a means to enhance their conductivity. Most interestingly, two different methodologies to deposit the enzyme onto the sensor surfaces were followed; strikingly different results were obtained depending on the family of pesticides under investigation. Furthermore, and towards the uniform application of the functionalization layer onto the SPEs’ surfaces, the laser induced forward transfer (LIFT) technique was employed in conjunction with CB functionalization, which allowed a considerable improvement of the sensor’s performance. Under the optimized conditions, the fabricated sensors can effectively detect carbofuran in a linear range from 1.1 × 10^−9^ to 2.3 × 10^−8^ mol/L, with a limit of detection equal to 0.6 × 10^−9^ mol/L and chlorpyrifos in a linear range from 0.7 × 10^−9^ up to 1.4 × 10^−8^ mol/L and a limit of detection 0.4 × 10^−9^ mol/L in buffer. The developed biosensor was also interrogated with olive oil samples, and was able to detect both pesticides at concentrations below 10 ppb, which is the maximum residue limit permitted by the European Food Safety Authority.

## 1. Introduction

Olive oil is one of the primary sources of added fat in the Mediterranean diet, with Spain (66%), Italy (14%) and Greece (14%) being the leading producers of olive oil in the European Union and worldwide. The European Union’s consumption of olive oil is estimated to be around 1.77 million metric tons (for 2019/2020, based on the European Commission data), and there is an increasing demand for high-quality and organic products [1]. In order to secure minimum harvesting losses, olive tree farmers use several agrochemicals, such as organophosphorus and carbamate pesticide, that may contaminate the produced olive oil, raising environmental and food safety concerns. Quantification of pesticides and their residues has thus become extremely important due to the concerns for public health raised due to their potential bioaccumulation, high toxicity and their long-term risk [2].

The current state-of-the-art methodologies for the determination of contaminants in olive oil are liquid (LC–MS/MS) and gas chromatography (GC–ECD) [3], methods that provide very accurate and reliable data for a large number of contaminants, including pesticide residues, but are costly and time-consuming. Fast and easy analytical tools, therefore, that can work alongside confirmatory methods such as chromatography coupled to mass spectrometry, are in high demand. 

Biosensors offer a promising alternative solution for the real-time and at the point-of-need analysis of olive oil. Specifically, for the detection of pesticides a number of different biorecognition molecules (antibodies [4,5], aptamers [6]) and biomimetic elements (imprinted polymers [7,8]) have been employed towards the development of affinity sensors [9] that rely on a number of different signal transduction principles (optical, piezoelectric and even mechanical).

Amongst them, electrochemical enzyme-based biosensors are especially suited for field applications since they are easy to manufacture, require low sample volumes (typically in the order of µL), have a short analysis time and have the ability to be integrated into compact and portable analysis devices [10]. Recently they have been used for the detection of pesticides in several real samples and complex matrices, such as tap water [11], vegetables [12], fruits, milk [13] and olive oil [14]. Their principle of operation relies on the inhibitory effects different pesticides have on enzymes. For example, carbamate and organophosphorus pesticides are known for their toxicity in mammals due to their ability to inhibit and thus inactivate the enzyme acetylcholinesterase, resulting in the accumulation of the neurotransmitter acetylcholine at the neuromuscular junctions, causing acute toxicosis [15]. This inhibition is translated into less thiocholine being produced by the hydrolysis of acetylthiocholine by AChE, which can be used for the evaluation of the enzyme’s activity. The concentration of thiocholine can be electrochemically determined, since it can be oxidized when current is applied, albeit at high overpotentials, which leads to high background noise since there is interference from other electroactive compounds [16]. In order to eliminate or reduce this interference, different types of redox mediators, such as Prussian blue, potassium ferricyanide [17] and cobalt phthalocyanine [18], can be used to lower the potential required for thiocholine oxidation. At the same time and in order to increase the conductivity and ultimately the sensitivity of the biosensing platform, several different nanomaterials have been exploited (nanotubes, nanoparticles) alone or in combination [19,20,21]. There is a plethora of publications describing the development of such functionalization protocols, which have been extensively reviewed elsewhere [22]. As an example, Liu et al. were the first to develop a carbon nanotube glassy carbon electrode for the low-current amperometric determination of enzymatically-produced thiocholine concentration [23], whereas Sun and Wang used Prussian blue as a redox mediator for the development of an amperometric biosensor towards the detection of organophosphate insecticides [24]. Zhou et al., on the other hand, introduced a combination of SnO_2_ nanoparticles and carboxylated graphene to modify glassy carbon electrodes for the detection of organophosphate and carbamate insecticides, at a low applied potential, achieving good reproducibility and low detection limits [25].

Nevertheless, and despite the advances made towards the sensitive detection of many different pesticides and pesticide residues, the increasing complexity and cost of the proposed modifications has prohibited their deployment in the field. To address the need for cost-effective enzyme-based biosensors without comprising on sensitivity or selectivity, Arduini et al., in 2010, was one of the first to pioneer the use of conductive nanostructured carbon black (CB) as an alternative to graphene and carbon nanotubes for the modification of screen-printed electrodes (SPEs) [26]. SPEs satisfy the need for highly reproducible and cost-effective substrates for electrochemical measurements, with the additional advantages of low cost of production, flexibility in design, small size and ease of electrode surface modification [27]. Moreover, CB is a commercially available by-product obtained by thermal decomposition of heavy petroleum residue [28]. As a substrate it mainly consists of elemental carbon and is mainly used in industry as a filler in plastics and elastomers; a colorant; a UV protectant; and an opaque and reinforcement material [29]. Moreover, some types of commercially available CBs are highly conductive [30] and have drawn researchers’ attention as materials for electrode modification and biosensor fabrication [31].

In this work, the effects that surface functionalization with CB has on the detection of an organophosphorus and a carbamate pesticide, chlorpyrifos and carbofuran respectively, were evaluated by comparing two different protocols for the deposition of AChE onto SPEs. Furthermore, and in attempt to improve on reproducibility of the sensor’s performance, the LIFT technique was employed for the modification of the electrodes. Finally, the optimized sensor fabrication protocols were used for the first time towards the ultra-sensitive detection of the two aforementioned pesticides in olive oil samples.

## 2. Results and Discussion

### 2.1. Choice of Substrate

**Figure 1:**
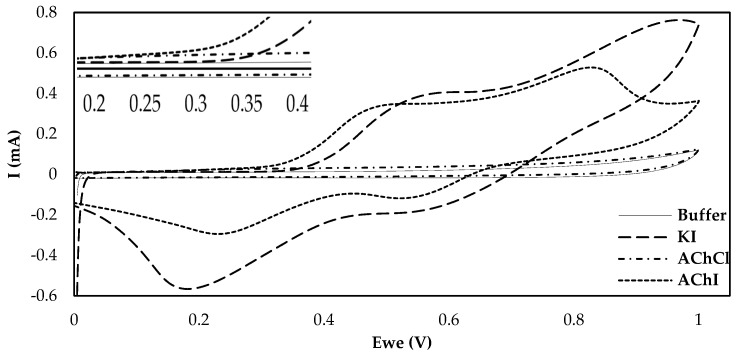
Cyclic voltammograms on CB/CS-modified carbon screen-printed carbon electrodes (SPEs) of phosphate buffer, 10 mM AChCl, 10 mM AChI and 10 mM KI at a scan rate of 50 mV/s. An enlarged image of the CVs at potentials between 200 and 400 mV is provided in the inset.

One of the most crucial considerations in the fabrication of an enzyme-based electrochemical biosensor is the choice of surface functionalization. CB, being able to enhance both the conductivity and the active surface over which the enzyme can be loaded has already been extensively studied in combination with various different enzymes, different ways of applying this material onto the electrode surface and different types of CB [16,31]. Nevertheless, the effects different substrates have on the current produced following their enzymatic catalysis by AChE have never been studied before on CB-modified electrodes. The majority of the fabricated AChE biosensors rely on the use of the chloride salt of acetylthiocholine. This is no coincidence, as the iodide salt is electrochemically active and can produce a false analytical signal that does not depend on the enzymatic action, due to its own oxidation. Nevertheless, and as Bucur et al. pointed out, the iodide salt of thiocholine can still be employed as a pseudosubstrate for AChE if the electrochemical behavior of the electrode towards thiocholine in the presence of iodide is evaluated [32]. The results in Figure 1 illustrate that the current produced upon the oxidation of enzymatically-produced thiocholine on functionalized electrodes drastically differs for AChCl and AChI. However, interrogation of the CB/chitosan (CS)-modified electrodes by CV in the presence of iodide ions (KI) reveals that, at overpotentials exceeding +350 mV, the recorded current is due to the oxidation of iodide and not of thiocholine. Thiocholine oxidation, on the other hand, is responsible for the increase in the anodic current when an applied potential between +200 and +300 mV is applied to the modified electrodes. The oxidation peaks for both salts coincide up to the potential of +250 mV, which was chosen as the applied potential in the amperometric measurements undertaken in the chronoamperometric measurements described in this manuscript. As far as the two different salts of acetylthiocholine are concerned and due to the fact that a cost-efficient solution that can potentially be commercialized was sought, iodide acetylthiocholine was chosen for all subsequent experimental measurements, as it is considerably cheaper in comparison to the chloride salt.

### 2.2. Optimization of Biosensor Fabrication

**Figure 2:**
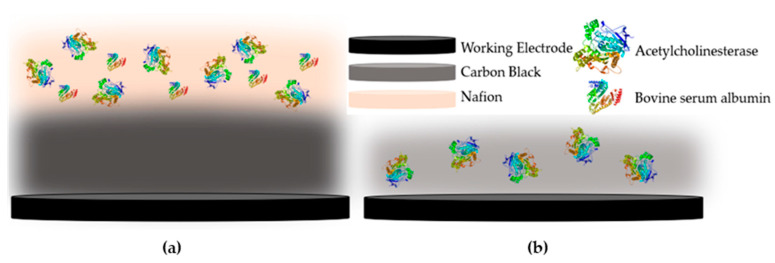
Graphical representation of the fabricated enzyme-based biosensor using (**a**) the multistep and (**b**) the one-step approaches.

Another aspect that greatly affects the performance of an enzyme-based biosensor is the way that the enzyme is deposited onto the surface of the electrode. Several different approaches have been put forward that result in its entrapment, physical adsorption or even covalent immobilization onto the sensor [33]. In the case of CB/CS enzyme-based sensors, the vast majority of researchers propose the covalent immobilization of the enzyme onto the CS mesh with the use of glutaraldehyde [34,35], with one notable exception. Talarico et al. developed an AChE-based biosensor for the detection of paraoxon, an organophosphorus pesticide, where CB dispersed in chitosan (CS) was used not only for the enhancement of the biosensor’s conductivity but as a means to entrap the enzyme onto the electrodes [36]. In this work, a direct comparison of these two methodologies was attempted for the first time. In the first approach, the enzyme was mixed at different concentrations with the CB/CS mixture and was drop-casted on the working electrode surface (denoted one-step). Alternatively, the enzyme was covalently immobilized onto the CB/CS-functionalized sensor with the use of glutaraldehyde (denoted multistep). A graphical representation of the two approaches to fabricating the enzyme-modified sensors is provided in Figure 2.

In addition, optimization of the immobilized enzymatic units is a crucial step to achieve a low detection limit, as low amounts of enzyme deposited onto the sensor surface result in low currents, whereas large amounts of enzyme do increase the product of the enzyme-catalyzed reaction, and hence, current intensity, but are less sensitive to low inhibitor concentrations. The responses, therefore, of the CB/CS/AChE sensors fabricated following both approaches and with different enzyme units deposited onto the surface, were tested prior to and upon incubation with three different concentrations of carbofuran.

**Figure 3:**
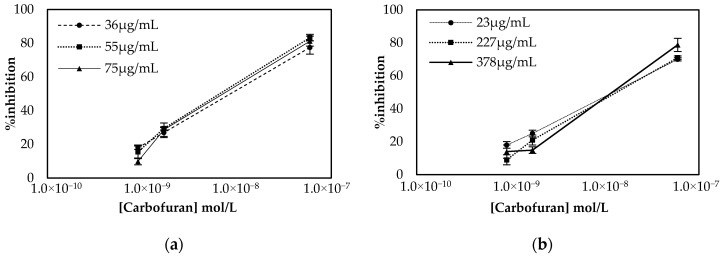
Optimization of enzyme concentration for sensors fabricated following the (**a**) multistep and (**b**) one-step approaches. Percentage inhibition of the enzyme was calculated following incubation of the biofunctionalized sensor with three different concentrations of carbofuran and recording the current amperometrically at an applied potential of +250 mV. All measurements were taken in triplicate.

The results presented in Figure 3 demonstrate that comparable percentage inhibitions (% inhibitions) are obtained with both approaches. However, considerable differences are observed in the units of enzyme required in the two different protocols. In the multistep approach, a steep increase in % inhibition is observed when the largest concentration of enzyme is employed. This indicates that a considerable number of enzymes lose their catalytic activity once covalently bound onto the surfaces, and this is why relatively large concentrations of the enzyme are required. On the contrary, in the one-step approach, reproducible results were obtained with 23 μg/mL of enzyme, due to the fact that the enzyme was entrapped in the biocompatible CS mesh. Based on these results, 75 μg/mL of AChE (150 ng of enzyme deposited onto the electrodes surface) was chosen for the realization of the multistep approach to fabricate the sensor, while 23 μg/mL (69 ng of enzyme) was used when the sensor was fabricated using the alternative one-step approach.

### 2.3. Measurement of Pesticide Inhibition of AChE in Standard Samples in Buffer

**Figure 4:**
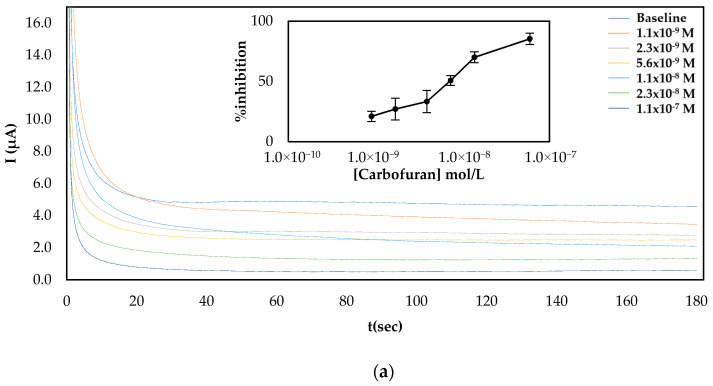

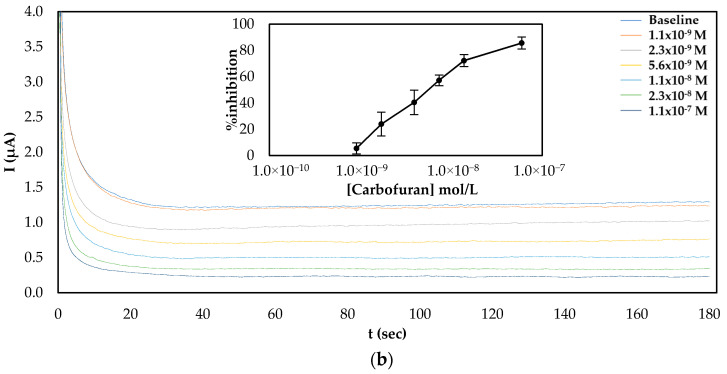
Characteristic amperograms of current recorded over time for the uninhibited enzyme (baseline) and following incubation with increasing concentrations of carbofuran in standard samples for CB/CS/AChE-modified electrodes fabricated following the (**a**) multistep and (**b**) one-step approaches at a constant applied potential of +250 mV. The graphs shown in the insets show the mean percentage inhibitions (% inhibitions) of different concentrations of carbofuran obtained from 5 different electrodes.

Since both fabrication approaches produced comparable results, both of them were subsequently employed for the establishment of a calibration curve of the % inhibition AChE with increasing concentrations of carbofuran and chlorpyrifos in standard samples. As far as carbofuran is concerned, a low limit of detection (LoD) was attained with both methodologies while the enzyme was inhibited over a wide dynamic range. Nevertheless, there are noticeable differences between the two fabrication approaches due to the different ways that the enzyme was deposited onto the surfaces (see Figure 4). For the multistep approach, the larger total amount of enzyme immobilized onto the sensors’ surfaces resulted in higher currents being recorded. Nevertheless, small concentrations of carbofuran cannot be as reliably detected as with the sensor fabricated following the one-step approach. 

**Figure 5:**
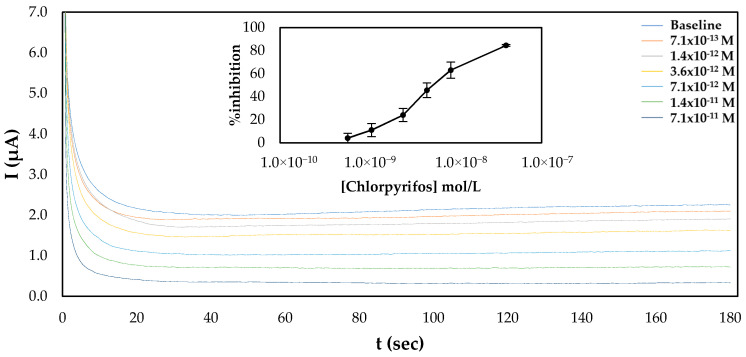
Characteristic amperograms of current recorded over time for the uninhibited enzyme (baseline) and following incubation with increasing concentrations of chlorpyrifos (standard samples) for CB/CS/AChE-modified electrodes fabricated following the one-step approach at a constant applied potential of +250 mV. The graph shown in the inset shows the mean % inhibitions of different concentrations of carbofuran obtained from 5 different electrodes.

For chlorpyrifos, both approaches to fabricate the sensor were also used. Surprisingly, a calibration curve of % inhibition of AChE with a range of different pesticide concentrations was established only for the sensors prepared following the one-step approach (Figure 5), as minimal inhibition of the immobilized enzyme onto sensors that were prepared following the multistep approach was observed (Figure S1). This discrepancy cannot be explained by the different mechanisms behind the inhibition of AChE by the two pesticides. It is known that the inhibition of AChE by organophosphorus pesticides such as chlorpyrifos is due to the phosphorylation of the serine hydroxyl in the enzyme active site. The phosphorylated enzyme is highly stable, and thus irreversibly inhibited; its inhibition depends solely on the chemical reactivity of the organophosphorus ester. By contrast, a good fit of the carbamate into the enzyme’s active site is essential for the formation of the enzyme–inhibitor complex prior to carbamylation, and thus crucial for its anticholinesterase activity [32]. If this was the reason behind the observed phenomenon, it would seem more reasonable not to be able to detect carbofuran with the sensor fabricated following the multistep approach, as the covalent immobilization of the enzyme through its amine groups on the glutaraldehyde-modified surface could potentially distort its tertiary structure, rendering impossible the formation of the enzyme–inhibitor complex. A plausible explanation that should be further investigated is that while the chemical reactivity of the phosphorus atom is of prime importance for anticholinesterase activity, steric properties sometimes have a strong effect on the anticholinesterase activity of an organophosphorus ester [32]. This means that although the immobilized enzyme is still capable of catalyzing the breakdown of acetylthiocholine, chlorpyrifos, due to its size and structure, is unable to enter the active site and thus exert its inhibitory effect on AChE. If this is true, this would appear to be specific to the particular means of electrode functionalization utilized here, which results in the formation of multiple bonds between AChE and the CB/CS mesh, as in other published articles where AChE was immobilized again through its amine groups with the use of either glutaraldehyde [37] or through the formation of amide bonds with the use of the EDC/NHS chemistry [38]—probably through fewer attachment points—chlorpyrifos could be detected by the covalently attached enzyme. Comparison of the apparent Km for the sensors fabricated following the one-step and multistep approaches reveals that the enzyme immobilized via glutaraldehyde (multi step approach) onto the electrode surfaces has roughly ten time less affinity for its substrate (Km = 2.56 mM) than the enzyme entrapped in the CS matrix (Km = 0.29 mM) (Figure S2), which further supports this hypothesis. Similar values for the apparent Km have been reported by other research groups—for example, AChE was entrapped in a chitosan matrix once (Km = 0.69 mM) [36] and crosslinked with glutaraldehyde in another study (1.1 mM) [39]. 

### 2.4. Improving on Surface Functionalization Reproducibility 

**Figure 6:**
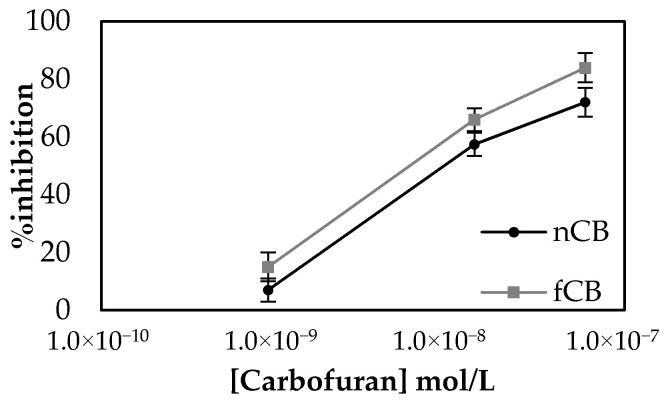
Percentage inhibitions achieved by non-functionalized CB (nCB)/CS/AChE sensors for three different concentrations of carbofuran versus the % inhibitions for the same concentrations of pesticide recorded with the use of functionalized CB (fCB)/CS/AChE sensors. The measurements were taken in triplicate.

In order to achieve reliable electrochemical measurements, the quality of surface functionalization is of paramount importance. In principle, the modifications introduced onto the working electrode surface must be evenly spread throughout and applied reproducibly each time the sensor is fabricated. One major obstacle in achieving the former is the fact that the CB dispersion within the CS matrix tends to be inhomogeneous and the CB itself quickly precipitates, even after vigorous vortexing. This is because most types of CB powders are mainly composed of pure (97%) elemental carbon according to the International Carbon Black Association (ICBA) [29]. However, there are other elements present, such as hydrogen, oxygen, nitrogen and sulfur. Treating CB with an acid or a base could increase the amount of oxygen present on the CB nanoparticles’ surfaces, and hence enhance hydrogen bonding with the surrounding medium. Several such attempts have been reported, whose primary goal was to improve on CB’s conductivity [40,41]. Herein, Redin’s protocol was followed to treat CB. As previously reported in the literature, negligible differences were observed when CB and functionalized carbon black (fCB) functionalized electrodes were characterized with the use of a redox pair (Figure S3). Nevertheless, the oxidation of enzymatically produced thiocholine onto fCB and CB functionalized electrodes did show a shift to slightly lower potentials and produced higher currents at +250 mV (Figure S4). Employment of the functionalized carbon black (fCB) to fabricate sensors using the one-step approach resulted in improved % inhibitions for the same pesticide concentrations in comparison to the sensors modified with the non-functionalized CB (nCB) (Figure 6). Most importantly, functionalized CB appeared to be considerably more hydrophilic than the non-functionalized one, which resulted in its better dispersion in the CS mesh and a more uniform suspension when mixed with the aqueous enzyme solution. This increase in hydrophilicity achieved with fCB was verified by contact angle measurements, where a decrease in the contact angle of the fCB/CS layer in comparison to that of the CB/CS was measured (Table 1). Moreover, SEM analysis of the surfaces further attests to the increased homogeneity of fCB in the CS mesh in comparison to non-functionalized CB (Figure S5). For all these reasons, fCB was used in all subsequent experimental procedures. 

Furthermore, and in order to improve on the amount of deposited material, the LIFT technique was used, which has been shown to deliver controlled amounts of biomolecules, inks and adhesive layers [42,43]. Spotting with the use of the LIFT technique introduces two further significant advantages in comparison to drop-casting. On the one hand, the amount of deposited material can be minimized, thereby reducing the cost of sensor fabrication [44], while at the same time high spatial resolution can be achieved [45], which renders it ideal as a technique for the biofunctionalization of sensor surfaces of minute dimensions.

**Figure 7:**
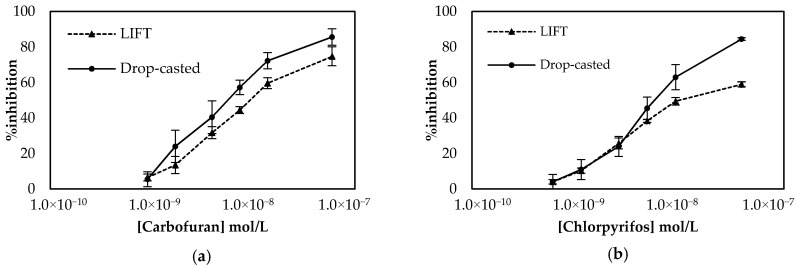
Comparison of the calibration curves for (**a**) carbofuran and (**b**) chlorpyrifos in buffer obtained using sensors functionalized with fCB/CS/AChE following the one-step approach. The sensors were prepared either with the LIFT technique of with the use of drop-casting. All measurements were acquired in triplicate.

Employment of the LIFT technique to functionalize the surface of the working electrode following the one-step approach resulted in the calibration curves shown in Figure 7. The sensors were prepared either with the LIFT technique of with the use of drop-casting for chlorpyrifos. The obtained results highlight the power of the LIFT technique to increase reproducibility as the variation between the measurements ranged from 1.4% to 3.8%, whereas for the sensors fabricated by drop casting the reproducibility was inferior as the variation between measurements ranged from 2.3% to 8.6%. This, in combination with the homogeneous dispersion of fCB, allowed the reliable detection of both pesticides, even at low concentrations. For carbofuran, a wide linear dynamic range was achieved for pesticide concentrations of 1.1 × 10^−9^ up to 2.3 × 10^−8^ mol/L, with a LoD equal to 0.6 × 10^−9^ mol/L (determined by the signal-to-noise approach, where an S/N larger than two was considered acceptable for estimating the detection limit) (2.4:1 for this concentration of carbofuran). For chlorpyrifos, the range of pesticide concentrations over which % inhibition exhibited a linear relationship was identical to the one obtained for carbofuran and extended from 0.7 × 10^−9^ to 1.4 × 10^−8^ mol/L. The achieved LoD in buffer was equal to 0.4 × 10^−9^ mol/L (2.8:1 signal-to-noise ratio).

**Figure 8:**
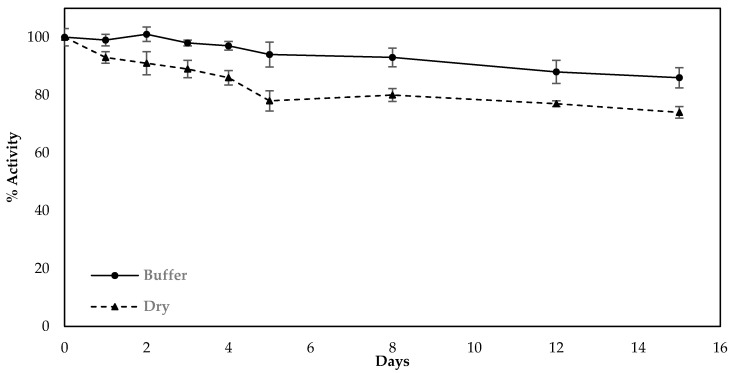
Decrease in activity of the sensors fabricated following the optimized protocol and upon storage at different conditions (dry or in buffer solution at 4 °C). The percentage decrease was calculated from the current recorded upon the interrogation of the sensors with freshly prepared substrate and how this compared with the current recorded when the sensors were interrogated as prepared. All measurements were taken in triplicate.

Finally, the stability of sensors fabricated with the use of the optimized protocol (modification of the electrodes in one-step by LIFT-spotted fCB/CS/AChE) was investigated over a period of two weeks. The fabricated sensors were stored either in dry conditions or in a buffered solution (1xPBS pH 7.4) at 4 °C and were amperometrically interrogated with freshly prepared substrate on a daily basis in the first five days and every two days from then onwards. The results indicate that when stored in a buffered solution at 4 °C, the biosensor retained 86.6% of its initial activity after 14 days, whereas the sensors stored dry deteriorated more rapidly and retained 74.3% of their initial activity for the same storage duration (Figure 8).

### 2.5. Measurements in Spiked Olive Oil Samples

**Figure 9:**
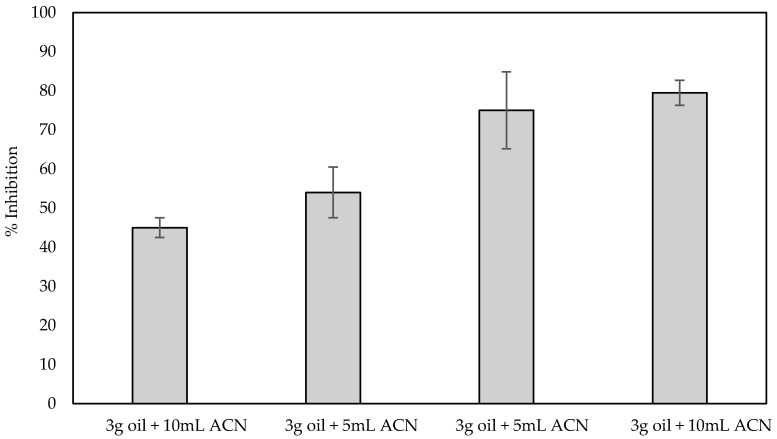
% Inhibition of AChE by 1 ppm carbofuran extracted from spiked olive oil. Four different ratios of sample:organic solvent were tested, and all measurements were recorded with fCB/CS/AChE-modified LIFT-spotted electrodes in triplicate.

In order to evaluate the applicability of the fabricated biosensor in spiked samples, sensors fabricated with the use of the optimized one-step approach were used for the detection of both carbofuran and chlorpyrifos in olive oil, while at the same time investigating the effect the matrix has on the % inhibition of the enzyme. For the pretreatment of olive oil and the extraction of analytes from it, a number of different protocols have been described that are based on the use of organic solvents. Nevertheless, most of them include a series of steps and techniques that are complex and time-consuming and cannot be easily transferred to a device that can be deployed in the field, including heating and centrifugation [46], centrifugation and solvent evaporation [47] and even the popular QuEChERS method [14]. Herein, pesticide extraction was attempted following a much simpler method that involves mixing of the olive oil sample with acetonitrile and the subsequent filtering of the supernatant through a PTFE membrane. Different ratios of olive oil:organic solvent were initially evaluated as to the effect the resultant filtrate has on the enzyme activity. It is worth noting that, irrespective of the ratio employed, a yellow tint could be discerned in all filtrates, indicating that filtration does not completely remove all the fat content from acetonitrile. Subsequent incubation of the fabricated sensors with the filtrates obtained following the four different protocols previously outlined (1:20 *v*/*v* ratio in buffer) resulted in the inhibition of AChE, that, independently of the olive oil:organic solvent ratio used, ranged from 6% to 16%, which is in full agreement with the data published by Loesche et al. where AChE inhibition was found to vary from 7% to 18% [48]. In order to account for this, all subsequent % inhibitions of the enzyme activity were calculated taking this into consideration; thus, we are reporting only the additional % inhibition of the enzyme caused by the extracted pesticide. To examine the extraction efficiency of the pretreatment protocols, olive oil samples were spiked with a range of different concentrations of carbofuran (10 ppb, 100 ppb and 1 ppm), and the filtrate was incubated with the fabricated sensors, as previously described for the control olive oil sample. For low analyte concentrations the % inhibition of the enzyme did not differ between the different pretreatment protocols (results not shown). Noticeable differences could only be observed when the oil samples were spiked with high concentrations of carbofuran (1 ppm). This is due to the fact that for high analyte concentrations, high extraction efficiencies are required, which can be achieved with increasing solvent volumes that, in turn, could increase the olive oil’s dispersion in the solvent and provide a larger interface between the two [49,50]. The results in Figure 9 reveal that the highest efficiency and reproducibility in the extraction of the pesticide from spiked olive oil is obtained when 10 g of olive oil is used and mixed with 10 mL of the organic solvent. In fact, the % inhibition achieved following this protocol (ca. 80%) is comparable with the % inhibition in a standard sample (ca. 75% for 1.1 × 10^−7^ mol/L (500 ppb)).

**Figure 10:**
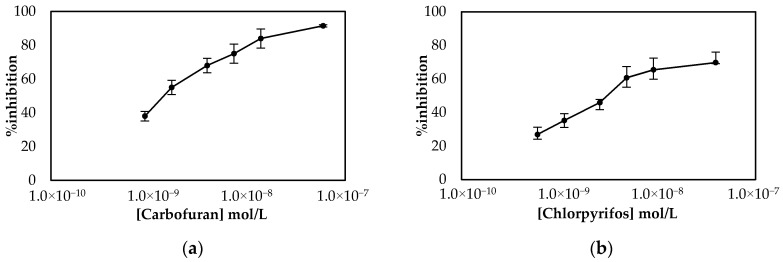
% Inhibition of AChE by a range of different concentrations of (**a**) carbofuran and (**b**) chlorpyrifos in pretreated spiked olive oil samples. The measurements were carried out in triplicate with LIFT-spotted fCB/CS/AChE modified electrodes.

Having optimized the pretreatment protocol, a calibration curve was then drafted for both pesticides using olive oil samples spiked with different concentrations of them (Figure 10). The obtained results illustrate that in pretreated spiked olive oil samples carbofuran can be detected over the same linear dynamic range as in standard solutions (1.1 × 10^−9^ mol/L up to 2.3 × 10^−8^ mol/L), but for chlorpyrifos the linear detection range in spiked olive oil samples is shorter and ranges from 0.7 × 10^−9^ to 0.7 × 10^−8^ mol/L. Furthermore, the % inhibition of AChE was found to be higher in pretreated spiked olive oil samples than the inhibition of the enzyme in standard solutions of the same concentration, even though the inhibition exerted by the matrix itself had already been accounted for and subtracted. Oujji et al. observed a similar behavior when spiked samples were studied [46] and it is a phenomenon that should be further investigated to evaluate the possibility that the pesticides along with the fatty acids remaining in the extract upon filtration exert a synergetic and additive inhibitory effect on AChE, as it has already been demonstrated for a number of chemical compounds [51,52]. Despite the fact, therefore, that considerably lower concentrations of the two pesticides could be detected in spiked olive oil samples in comparison to standard solutions, it would not be valid to report lower LoDs than the ones achieved in buffer (Figure 7) due to the uncertainty as to the underlying causative reason for the observed inhibition. Lastly, the % inhibition of the enzyme by chlorpyrifos reaches a plateau at concentrations higher than 0.7 × 10^−8^ mol/L, which is not the case when the fabricated sensor is interrogated with identical concentrations of the pesticide in standard solutions. This could be explained taking into consideration that chlorpyrifos does not readily dissolve in acetonitrile (that is why a stock solution was initially prepared in heptane, from which spiked olive oil samples were prepared), and hence it was not extracted as efficiently as carbofuran from the spiked olive oil samples with the optimized pretreatment protocol. Furthermore, chlorpyriphos is considerably less soluble in aqueous solution (solubility in water, 1.4 mg/L at 25 °C) [53]. By contrast, the solubility of carbofuran is 351 mg/L at 25 °C [54].

The sensitivity in pesticide detection achieved with the fabricated sensor permits the detection of both pesticides in spiked olive oil samples at concentrations below the MRL (maximum residue limits) set by the EFDA, which are 0.01 ppm for both pesticides (2.3 × 10^−9^ mol/L for carbofuran and 1.4 × 10^−9^ mol/L for chlorpyrifos) (Part A of Annex I to Regulation 396/2005), and compares well with other published work where far more complex and cost-inefficient matrices for enzyme immobilization have been proposed (Table 2).

## 3. Materials and Methods

### 3.1. Materials

Acetylcholinesterase (AChE from electric eel), acetylthiocholine iodide (AChCl), acetylcholine chloride (AChI), 5,5′-dithio-bis-(2-nitrobenzoic acid) (DTNB), potassium hexacyanoferrate(III) (K_3_Fe(CN)_6_) and potassium hexacyanoferrate(II) trihydrate (K_4_[Fe(CN)_6_]·3H_2_O), medium molecular weight chitosan (CS) glutaraldehyde, Nafion^™^ (perfluorinated ion-exchange resin, 5% *v*/*v* solution in lower alcohols/water), bovine serum albumin (BSA), carbofuran (PESTANAL^®^, analytical standard), chlorpyrifos (PESTANAL^®^, analytical standard), acetonitrile, n-heptane, acetic acid and nitric acid were purchased from Sigma Chemical Company (St. Louis, MO, USA). Conductive carbon black (CB) (Vulcan XC 72R) was kindly provided by Cabot Corporation’s representatives in Greece (RAWCHEM), having an average particle size of 50 nm and typical bulk density of 6 lbs/ft^3^. PTFE Whatman syringe filters with a pore size of 0.45 μm where obtained from Sigma Chemical Company (ST. Louis, MO, USA). Disposable SPEs (Carbon DRP110) consisting of carbon ink based working electrode (4 mm diameter), a carbon counter electrode and an Ag reference electrode and a cable connector for screen-printed electrodes (DRP-CAC4MMH) were purchased from Metrohm DropSens (Oviedo, Asturias, Spain). Phosphate Buffer (50 mΜ, 0.1 M KCl, pH 7.4) was used for all the experimental measurements unless otherwise stated. Extra virgin olive oil was provided by MINERVA S.A, Greece. All solutions were prepared with Milli-Q water (18.2 MΩ∙cm).

### 3.2. Apparatus

Cyclic voltammetry (CV) and chronoamperometry (CA) were performed using a BioLogic potentiostat/galvanostat instrument (Seyssinet-Periset, France). Contact angles were measured. A side view imaging setup, consisting of high-speed camera (Mini AX-100, Photron by Photron Europe Limited, Buckinghamshire, UK), coupled with a 50× microscope objective (Edmund) and a standard LED source (Thorlabs LEDD1B) placed opposite of the camera for illumination purposes, was built for the contact angle measurements of the all in one solution hydrophilic/ hydrophobic droplets on the surfaces of the electrodes. For the colorimetric determination of the enzymatically produced thiocholine and for the inhibition of AChE in solution by carbofuran and chlorpyrifos a Tecan SPARK10M multimode microplate reader was employed.

### 3.3. Electrode Functionalization with CB and fCB

An aqueous solution of 0.05 M HCl was prepared and heated at 90 °C. Sufficient CS was added and dissolved under stirring for approximately 30 min, resulting in a final concentration of 0.05% *w*/*v*. Thereafter, 3 mg/mL of CB was added to the CS solution, which was subsequently allowed to cool to room temperature. The mixture was left overnight under stirring in order to achieve the best possible dispersion. For the functionalized CB (fCB) the protocol published by Ibáñez-Redín et al. was followed [40]. Briefly, 50 mg of CB was dispersed in 100 mL of concentrated sulfuric and nitric acid in a ratio of 1:1(*v*/*v*) and stirred for 30 min in room temperature. The dispersion was then filtered and the fCB was washed and re-dispersed in ultrapure water and filtered several times, until the supernatant washing water reached a pH of 7.0. Functionalized CB was subsequently dried in an oven at 120 °C for 12 h. The fCB was then dispersed in the CS solution at the same concentration as the non-functionalized CB. Prior to their usage both the CB/CS and fCB/CS solutions were vigorously vortexed, drop-casted onto the working electrode surface (2 × 3 μL) and allowed to dry at room temperature for 1 h. The modified electrodes were subsequently characterized by cyclic voltammetry (CV) using ferri/ferrocyanide redox couple (1 mM in equimolar concentrations) in phosphate buffer at a 50 mV/s scan rate from 0.6 to −0.2 V. To determine the hydrophilicity of the functionalized surfaces, contact angle measurements were performed.

### 3.4. SPE Characterization with Enzymatically-Produced Thiocholine

In order to establish the potential at which thiocholine gets oxidized on the functionalized SPEs, cyclic voltammetry with enzymatically-produced thiocholine was performed. For its production, both AChCl and AChI were used as substrates, while solutions were also prepared fresh. For this purpose, 273 μg/mL of the enzyme was mixed with 10 mM of either substrate in phosphate buffer (50 mΜ, 0.1 M KCl, pH 7.4), stirred and left at room temperature for 2 h. After 2 h, acetylthiocholine hydrolysis was complete. The concentration of the produced thiocholine was estimated spectrophotometrically by Ellman’s method by mixing 450 μL of phosphate buffer solution (0.1 M, pH = 8), 50 μL of 0.1 M DTNB, and 5 μL thiocholine solution (diluted 1:100 in water) in spectrophotometric cells. The absorbance was then measured at 412 nm, and the real concentration was evaluated by using the Lambert–Beer law with the known molar extinction coefficient of TNB (ε = 13,600 M^−1^ cm^−1^) [64]. Cyclic voltammograms of the enzymatically produced thiocholine (10 mM) on CB functionalized electrodes were then acquired by cycling the potential from 0.00 to +1.00 V at a scan rate of 50 mV/s.

### 3.5. Fabrication of the Biosensor

For the modification of the functionalized SPEs with enzyme, two different methodologies were followed. The enzyme was either mixed in the CB/CS dispersion (one-step procedure) or was covalently immobilized onto the CS/CB-modified SPEs (multi-step procedure). For the former, the enzyme was diluted in phosphate buffer and added in the CB/CS dispersion in a 1:2 *v*/*v* ratio, resulting in three different final dilutions (23, 227 and 378 μg/mL). The AChE/CB/CS mixture was thoroughly vortexed and 3 μL was drop-casted directly onto the working electrode and left to dry at room temperature. For the multi-step modification of the electrodes, 3 μL οf the CB/CS mix was drop-casted onto the working electrode (2 sequential additions) and left to dry prior to the application of 2 μL of glutaraldehyde (0.25% *v*/*v* in water). Finally, AChE was allowed to bind covalently onto the electrode following application of 2 μL of the enzyme mixture (3% BSA, 0.1% Nafion, AChE dissolved in PBS buffer in equal ratios by volume) onto the glutaraldehyde-modified CB/CS-functionalized SPEs. The final concentrations of the enzyme used in the multi-step procedure were 36, 55 and 75 μg/mL.

### 3.6. Measurement of AChE Inhibition by Chlorpyrifos and Carbofuran

The inhibition of AChE by the two pesticides was evaluated both colorimetrically in solution and electrochemically with the use of amperometrical measurements of the CB/CS/AChE-modified SPEs. In both cases the percentage inhibition (%I) was calculated with the following formula:

I% = ((I_0_ − I_1_)/I_0_) × 100 (1)

where I_0_ is the activity of the uninhibited enzyme and I_1_ that of the enzyme upon incubation with different concentrations of the two pesticides. Stock solutions of the two pesticides were prepared by dissolving 2 g/L chlropyrifos in n-heptane and 2 g/L carbofuran in acetonitrile. Dilutions of the stock pesticide solutions were prepared in acetonitrile for both pesticides.

For the colorimetric determination of enzyme inhibition, aliquots of 30 μL from the pesticide solution were incubated with 300 μL AChE (273 μg/mL) in phosphate buffer for 20 min at room temperature. Then 120 μL of ATChI (2.5 mM) and 150 μL of DTNB (2 mM) were added to the enzyme and were allowed to incubate for another 20 min. Its absorbance was then recorded in triplicate at 412 nm. Assays in the absence of inhibitor (blank) served as control.

For the electrochemical determination of the percentage inhibitions of the two pesticides, the current intensity at the CB/CS/AChE-modified SPEs (fabricated using both the one-step and the multi-step approach) was recorded at a constant applied voltage of +0.25 mV following stabilization for 5 min in phosphate buffer solution. Pesticide detection was done in a three-step procedure as follows: first, the initial response of the electrode to 2.5 mM ATChI (60 μL) was recorded (baseline); then the electrode was incubated in a solution containing a known concentration of pesticide for 20 min; and finally, the residual response of the electrode was recorded again. The pesticide solutions were prepared by mixing a range of different concentrations of carbofuran or chlorpyrifos in acetonitrile with phosphate buffer (1:20 *v*/*v* ratio). To account for the inhibition to the enzyme activity caused by acetonitrile present in the pesticide solutions, 5% *v*/*v* ACN was added in all of the used solutions (buffer for stabilization, substrate dissolved in buffer). The measured signal corresponded to the difference of current intensity between the baseline and that measured upon incubation with the pesticide solutions. To establish the calibration curves of enzyme inhibition over a range of pesticide concentrations both in standard samples and real samples, the same fabricated sensor was incubated with increasing concentrations of the pesticide following the procedure outlined above.

### 3.7. Laser Induced Forward Transfer (LIFT) Technique for SPEs Surface Modification

LIFT experiments were carried out using a pulsed Nd:YAG (neodymium-doped yttrium aluminum garnet) laser (355 nm wavelength, pulse duration of 10 ns) and a high power imaging micromachining system [65]. After the laser beam exits the laser source, it passes through an optical setup to determine the shape and the size of the laser beam which will irradiate a donor substrate that carries the material to be deposited. The donor substrate consisted of a layer which absorbed the laser pulse and a transparent carrier. On top of the absorbing layer, a thin film (approximately 5 μm width) of the desired liquid material is coated. During the printing process the donor substrate is placed parallel and in close proximity to a receiver substrate (300 μm donor–receiver distance). Following the irradiation of the donor substrate, a high-pressure air bubble is created in the interface between the absorbing layer and the liquid to be deposited. As the bubble expands, a liquid jet is created which results to the deposition of a droplet at the receiver substrate. By controlling the irradiation conditions and the characteristics of the laser pulse, the size of the deposited droplets can be tuned.

**Figure 11:**
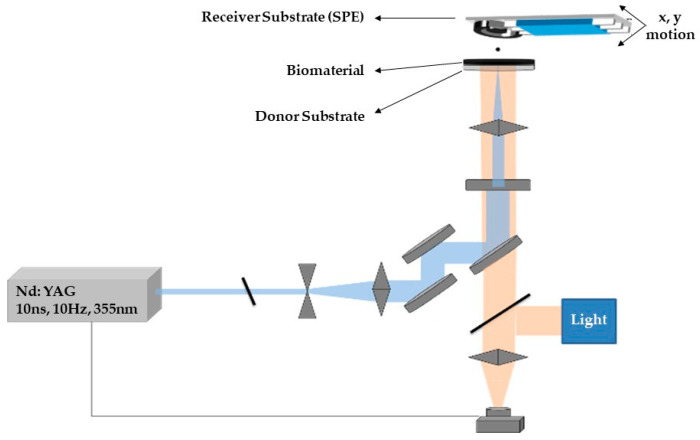
Schematic representation of the laser induced forward transfer (LIFT) experimental setup.

The deposition of the CB/CS/AChE or fCB/CS/AChE mixture (one-step procedure) was carried out by means of a laser printing micromachining system, as previously published [66], depicted in Figure 11. A LabVIEW program was used in order to synchronize the x–y motion with the laser. The laser beam was attenuated and expanded to irradiate an 80 × 80 µm square mask. Consequently, a 15× microscope objective lens was used to focus the laser beam on a donor substrate. The donor substrate consisted of a sacrificial layer (Ti film) on quartz plate, onto which a liquid thin film of the biomaterial to be deposited was coated. The transfer was carried out in such way that each droplet was deposited by a single pulse and a 20 × 20 array resulted in a continuous enzyme film (3 × 3 mm) on the electrode, which was placed 300 µm above the donor substrate. The fabricated sensors were characterized amperometrically, as described in the previous section, and their morphology was determined by scanning electron microscopy (FESEM Nova NanoSEM 230, by FEI Europe, Eindhoven, The Netherlands).

### 3.8. Real Sample Analysis

For the determination of pesticide concentrations in olive oil, samples were spiked with different concentrations of carbofuran or chlorpyrifos. The olive oil samples used were certified pesticide-free by the supplier that had previously analyzed them by an Oils and Fats Accredited LC-QTOF—GC-MS/MS using the Modified Method of analysis, code number O.B.05.48 (EN 15662:2018 and SANTE/11813/2017 of the European Commission). For both pesticides, a stock solution was prepared in olive oil at a concentration of 0.02 g/L from which olive oil samples of different concentrations were made (5 up to 500 ppb). For the extraction of the pesticide, olive oil was mixed with acetonitrile at different ratios and vigorously agitated for 5 min to form an emulsion. The latter was left undisturbed for 15 min, so that the two phases separate. Subsequently, 1 mL of the supernatant was obtained using a syringe and filtered through a PTFE/L 0.45 μm syringe filter. The filtrate was mixed with phosphate buffer (1:20 ratio) and used to electrochemically determine the percentage inhibition of AChE by the extracted pesticide.

## 4. Conclusions

In this work, a facile and low-cost surface functionalization approach was followed towards the fabrication of an amperometric acetylcholinesterase-based sensor for the detection of organophosphorus and carbamate pesticides. The method relies on the use of CB suspended in a CS matrix as a cost-efficient nanomaterial to enhance both the sensor’s conductivity and the active surface over which the enzyme can be deposited Extensive investigation into the parameters that affect the performance of the fabricated sensor led to the sensitive detection of both carbofuran and chlorpyrifos, while the achieved LoDs compare favorably with those attained by considerably more complex biosensing platforms. Interestingly, the functionalization of CB by acid treatment significantly improved its dispersity into the CS matrix, which permitted the more uniform and reproducible functionalization of the sensor surfaces. Furthermore, it was found that the inhibition the two different pesticides exert on the enzyme depends upon the method used to deposit acetylcholinesterase onto the sensor surfaces, which should be further investigated with the use of different pesticides. Interrogation of the fabricated sensor with spiked olive oil samples, following a simple extraction protocol, allowed the detection of both pesticides at levels compatible with the MRLs set by current European legislations, but also emphasized the inhibitory effect of fats on acetylcholinesterase. The obtained results highlight the synergetic inhibitory effect of fats and pesticides which should be further evaluated in the future using oils of different fatty acid compositions along with different pesticides in order to clearly elucidate the mechanism behind acetylcholinesterase inhibition.

## Figures and Tables

**Table 1:** Contact angle measurements of fCB/CS and nCB/CS modified electrodes.

CB Type	θ_C_ (°)	CA Error
fCB	7.84	0.910
nCB	13.79	0.540

**Table 2:** AChE-based biosensors for determination of carbamate and organophosphorus pesticides.

Electrode Material/ Immobilization Matrix	LOD in Buffer (pΜ)	Real Samples Tested	Reference
**Carbofuran**			
PAMAM-Au/CNTs/GCE	4 × 10^3^	Onion, Lettuce, Cabbage	[55]
NF/CS-PB-MWCNTs-HGNs/AuE	25 × 10^2^	Cabbage, Lettuce, Leek	[56]
NF/CS/NiONPs-CGR-NF/GCE	0.5	Apple, Cabbage	[57]
CB/AgNPs/GCE	10^2^	Peanut	[58]
CB/Pillar[5]arene	20	Peanut, Beetroot	[59]
CB/CS	6 × 10^2^	Olive Oil	This work
**Chlorpyrifos**			
Fe_3_O_4_NPs/MWCNT/Au electrode	10^2^	Tap Water, Milk	[60]
ZnS and poly(indole-5-carboxylic acid)/Au	1.5 × 10^2^	Tap Water	[38]
PANI/CNT wrapped with ssDNA/Au	1.0	River Water	[37]
MWCNTs/SnO_2_/CS-SPE	5 × 10^4^	Cabbage, Lettuce, Leek, Pak choi	[61]
MWCNTs/IL/SPE	5 × 10^4^		
Bromothymol blue doped sol–gel film	11 × 10^4^	Water	[62]
ZrO2/ERGO	0.1	Water	[63]
CB/CS	4 × 10^2^	Olive Oil	This work

AchE: acetylcholinesterase, PAMAM: poly(amidoamine), Au: gold, CNTs: carbon nanotubes, GCE: glass carbon electrode, NF: nafion, CS: chitosan, PB: Prussian blue, MWCNTs: multi-wall carbon nanotubes, HGNs: hollow gold nanospheres, AuE: gold electrode, NiONPs: nickel oxide nanoparticles, CGR: carboxylic graphene, CB: Carbon black, AgNPs: silver nanoparticles, FeO4NPs: magnetite nanoparticles, PANI: polyaniline, IL: ionic liquid, SPE: screen-printed electrode, ERGO: electrochemical reduced graphene oxide.

## Supplementary Materials

The following are available online.
