# Supplementary material for: Facile and Low-Cost SPE Modification Towards Ultra-Sensitive Organophosphorus and Carbamate Pesticide Detection in Olive Oil
Source: Molecules. 2020 Oct 28;25(21):4988. doi: 10.3390/molecules25214988 (PMC7672650; doi:10.3390/molecules25214988)
Supplement: Supplementary file 1 [file molecules-25-04988-s001.zip › molecules-911068-supplementary/molecules-911068-supplementary-for XML.pdf]

Article

Facile and Low-Cost SPE Modification Towards Ultra-Sensitive Organophosphorus and Carbamate Pesticide Detection in Olive Oil

Dionysios Soulis [1], Marianna Trigazi [1], George Tsekenis [1], Chrysoula Chandrinou [2], Apostolos Klinakis [1] and Ioanna Zergioti [2,*]

[1] Biomedical Research Foundation of the Academy of Athens, Soranou Ephessiou 4, 11527 Athens, Greece; dsoulis1@gmail.com (D.S.); mtrigazi@gmail.com (M.T.); gtsekenis@bioacademy.gr (G.T.); aklinakis@bioacademy.gr (A.K.)

[2] School of Mathematical and Physical Sciences, Physics Department, National Technical University of Athens, Heroon Polytehneiou 9, 157 80 Athens, Greece; cchandr@mail.ntua.gr

* Correspondence: zergioti@central.ntua.gr

Academic Editor: Gregorio F. Ortiz
Received: 9 August 2020; Accepted: 23 October 2020; Published: date

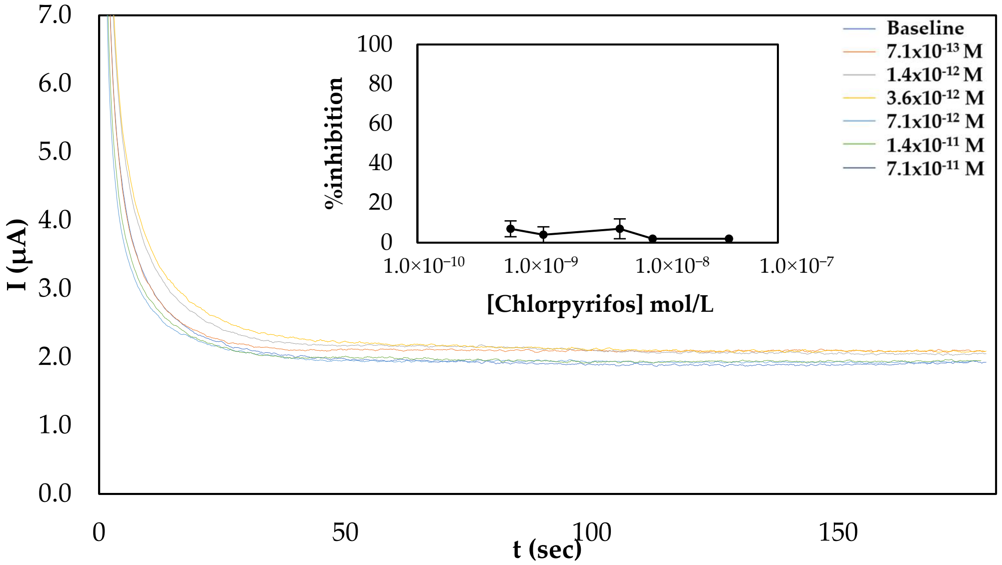

Figure S1. Characteristic amperograms of current recorded over time for the uninhibited enzyme (baseline) and following incubation with increasing concentrations of chlorpyrifos (standard samples) for CB/CS/AChE-modified electrodes fabricated following multistep approach at a constant applied potential of +250mV. The graph shown in the insets show the mean % inhibition of different concentrations of chlorpyrifos obtained from 5 different electrodes.

molecules

MDPI

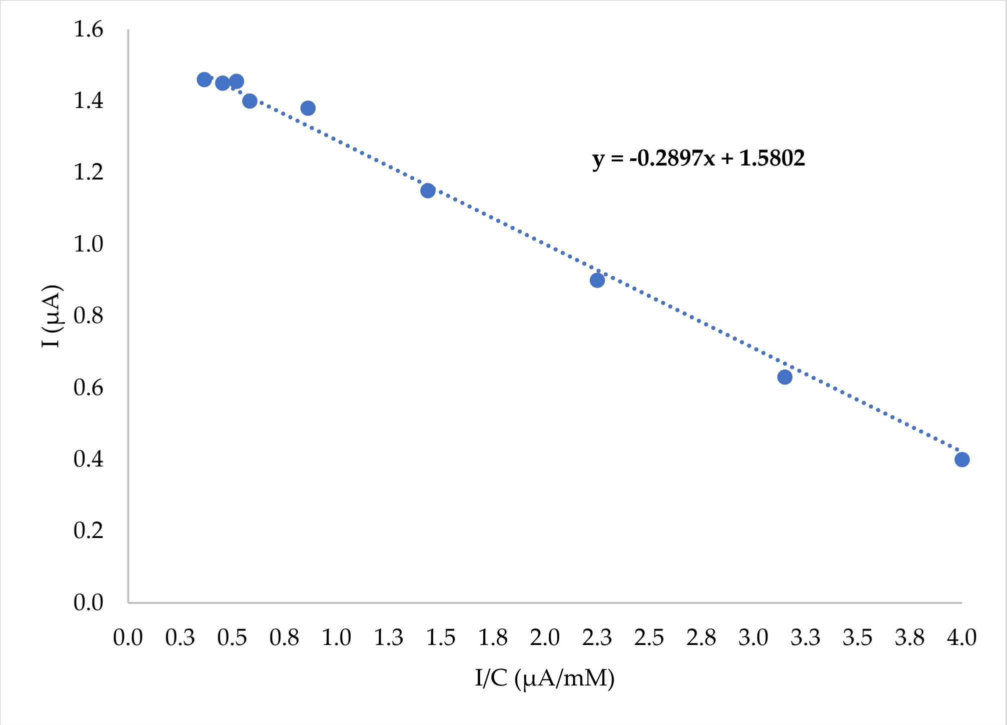

(a)

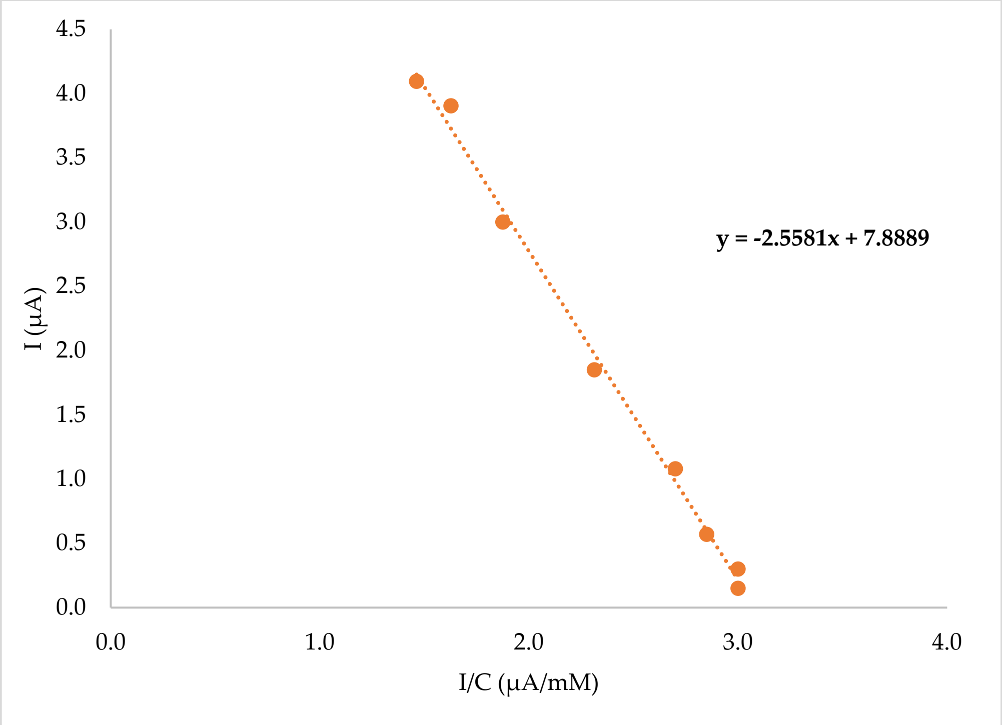

(b)

Figure S2. Eadie–Hofstee diagram for the Eadie-Hofstee plot for the determination of the apparent Michaelis-Menten constant Km of the a) entrapped AChE on sensors fabricated using the one-step approach and b) crosslinked enzyme on sensors fabricated using the multistep approach.

molecules
MDPI

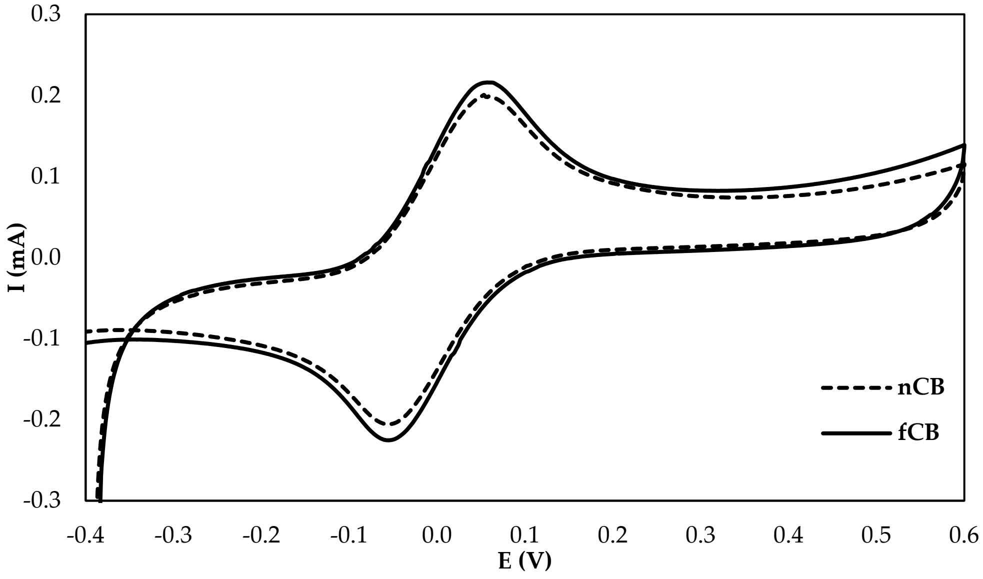

Figure S3. Cyclic voltammograms of 5mmM $K_3Fe(CN)_6/K_4Fe(CN)_6$ in 1xPBS on CB/CS-modified carbon SPEs and fCB/CS-modified carbon SPEs. Scan rate 50mV/sec.

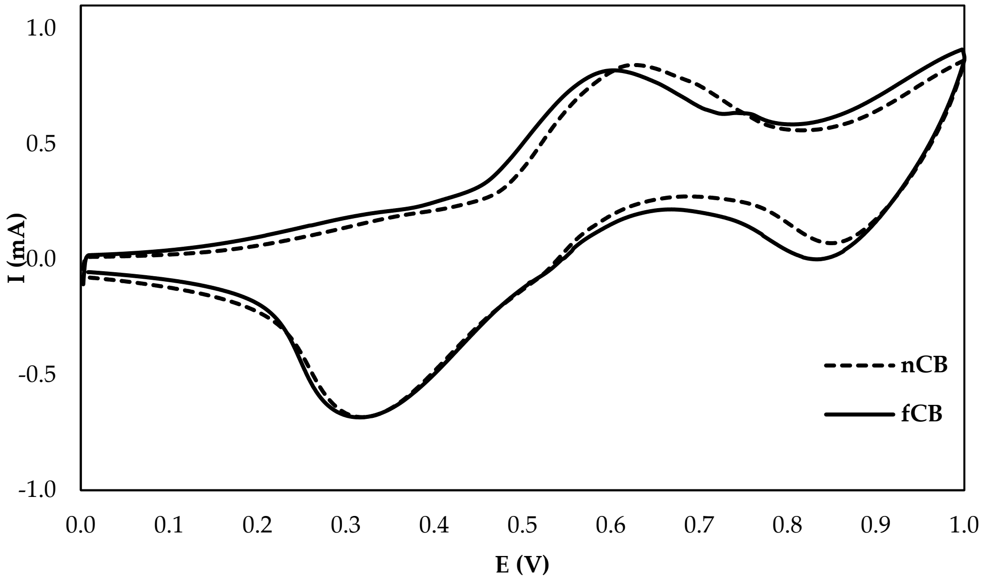

Figure S4. Cyclic voltammograms of 10mmM AChI on CB/CS-modified carbon SPEs and fCB/CS-modified carbon SPEs at a scan rate of 50mV/sec.

molecules

MDPI

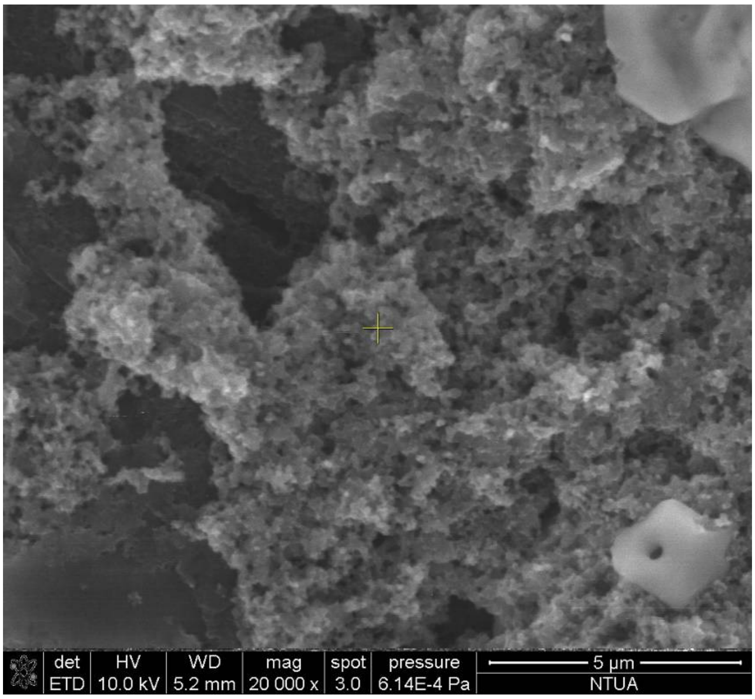

(a)

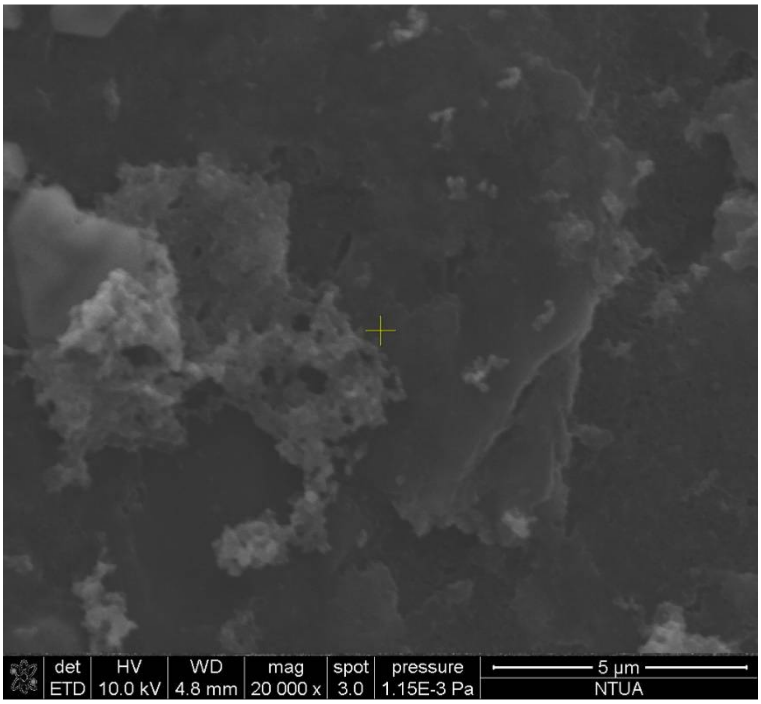

(b)

Figure S5 SEM images for electrode surfaces modified with (**a**) non-functionalized CB and (**b**) functionalized CB. In both cases the CB particles were dispersed in a CS mesh.
